# Cluster of *S. maltophilia* among patients with respiratory tract infections at an intensive care unit

**DOI:** 10.1016/j.infpip.2020.100097

**Published:** 2020-09-30

**Authors:** Maria Gideskog, Jenny Welander, Åsa Melhus

**Affiliations:** a Department of Infection Control and Hygiene, Linköping University Hospital, Linköping, Sweden; b Department of Clinical Microbiology, Linköping University Hospital, Linköping, Sweden; c Department of Medical Sciences/Section of Clinical Microbiology, Uppsala University Hospital, Uppsala, Sweden

**Keywords:** *S. maltophilia*, respiratory tract infection, calorimeter, sink

## Abstract

**Background:**

Stenotrophomonas maltophilia is associated with respiratory tract infections in immunocompromised patients, and it has emerged as an important nosocomial pathogen, with admission to intensive care units (ICUs) and ventilators as recognized risk factors.

**Aim:**

To describe the investigation of a sudden increase in patients with pneumonia caused by *S. maltophilia* at a Swedish ICU and the control measures taken.

**Methods:**

Lower respiratory tract cultures from patients admitted to the ICU were obtained, and environmental cultures were collected from sink drains and medical equipment. Isolates identified as *S. maltophilia* were subjected to antibiotic susceptibility testing and whole genome sequencing (WGS).

**Findings:**

A total of 17 *S. maltophilia* isolates were found (four from patients and 13 from the environment). The WGS identified two outbreak clones, sequence type (ST) 361 and ST138, and seven unique ones. Most likely, the outbreak clones originated from two sinks, and transmission was enhanced by a calorimeter. After changing the sink and calorimeter routines, no more cases were registered.

**Conclusion:**

Acquisition of *S. maltophilia* from the hospital environment appears to be easy, especially if water is involved. To control this bacterium, better knowledge of its transmission routes in hospital environments is required.

## Introduction

*Stenotrophomonas maltophilia* is a ubiquitous, non-fermenting Gram-negative rod with essential functions in plant ecosystems. Like *Acinetobacter* spp., it has generally been regarded as an opportunist, but in later years it has emerged as an important pathogen in hospital environments. It is mainly associated with respiratory tract infections in immunocompromised patients and bloodstream infections in neutropenic patients. It can, however, cause other serious infections, including meningitis, endocarditis and infections in bone, skin and soft tissues. Fatalities are not uncommon and admission to an intensive care unit (ICU) and ventilator use are recognized risk factors [[1], [2], [3]].

The productions of broad-spectrum beta-lactamases, efficient multidrug efflux pumps, low outer membrane permeability and a high ability to acquire resistance, renders *S. maltophilia* resistant to a broad array of antibiotics [2]. Trimethoprim-sulfamethoxazole is the only drug with breakpoints for *S. maltophilia*, according to the European Committee on Antimicrobial Susceptibility Testing (EUCAST), but therapy failures and resistance development during therapy occur [4]. Treatment of severely ill patients can therefore be a challenge.

Apart from its multiresistance, the bacterium is characterized by its ability to form biofilms on various abiotic and biotic surfaces [2]. In the hospital environment, sinks and drains with stagnant water constitute a high risk for contamination of *S. maltophilia*. Aerosols from sinks may contaminate medical devices used in the daily care of patients, and bacteria can thereby be transmitted to vulnerable patients [1,5].

A wide range of molecular genotyping methods can be used to identify the genetic relatedness of bacterial isolates and map their transmission routes. In contrast to traditional methods, whole genome sequencing (WGS) has an ultimate resolution power by permitting discrimination between closely related isolates through comparison of single nucleotide polymorphisms (SNPs) [6]. This feature can be useful when investigating clinical isolates of *S. maltophilia* [7,8], a species with a high genetic diversity.

The objective of this study was to describe the investigation performed to elucidate the background to a sudden increase in isolation frequency of *S. maltophilia* in patients with pneumonia at an ICU at Linköping University Hospital, Sweden. Furthermore, a description of the measures taken to control the dissemination of the bacterium is given.

## Methods

### Setting

Linköping University Hospital is the only tertiary care hospital in southeast Sweden. The involved ICU offers a total of eight beds. All rooms have a sluice, and there is a sink in each sluice and one in each patient room. The sinks are used for hand washing, patient care, cleaning of various medical devices, etc.

### Epidemiological investigation

**Figure 1:**
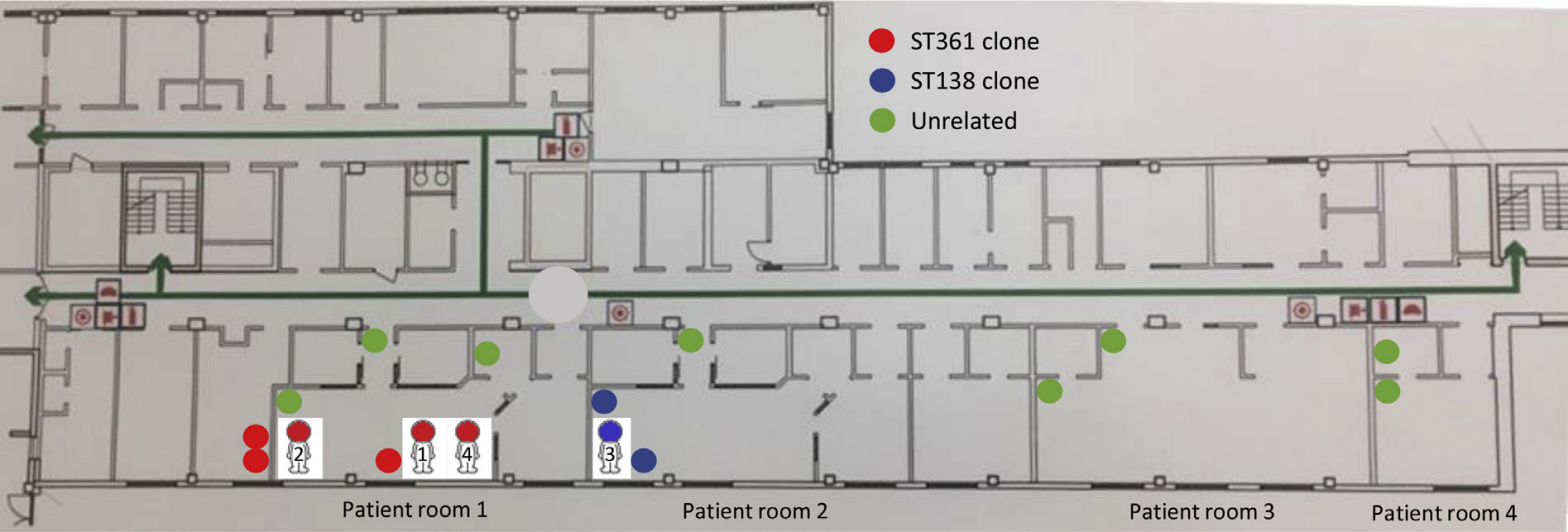
Drawing of the intensive care unit where the clustering of *S. maltophilia* took place. Patients and environmental sites culture-positive for the bacterium are marked with different colours depending on sequence type. (For interpretation of the references to colour in this figure legend, the reader is referred to the Web version of this article.)

In October 2018, three patients admitted to an ICU at Linköping's University Hospital showed growth of *S. maltophilia* in samples from their lower respiratory tracts within just a couple of days. The index patient (Patient 1) was culture positive for the bacterium prior to the admittance to the ICU. This patient shared the same room (room 1) with Patient 2, whereas the third patient (Patient 3) was located in the room next-door (room 2). For more details, see Figure 1. Due to the bacterial findings and the close proximity of the three patients in space and time, an epidemiological investigation was initiated.

**Figure 2:**
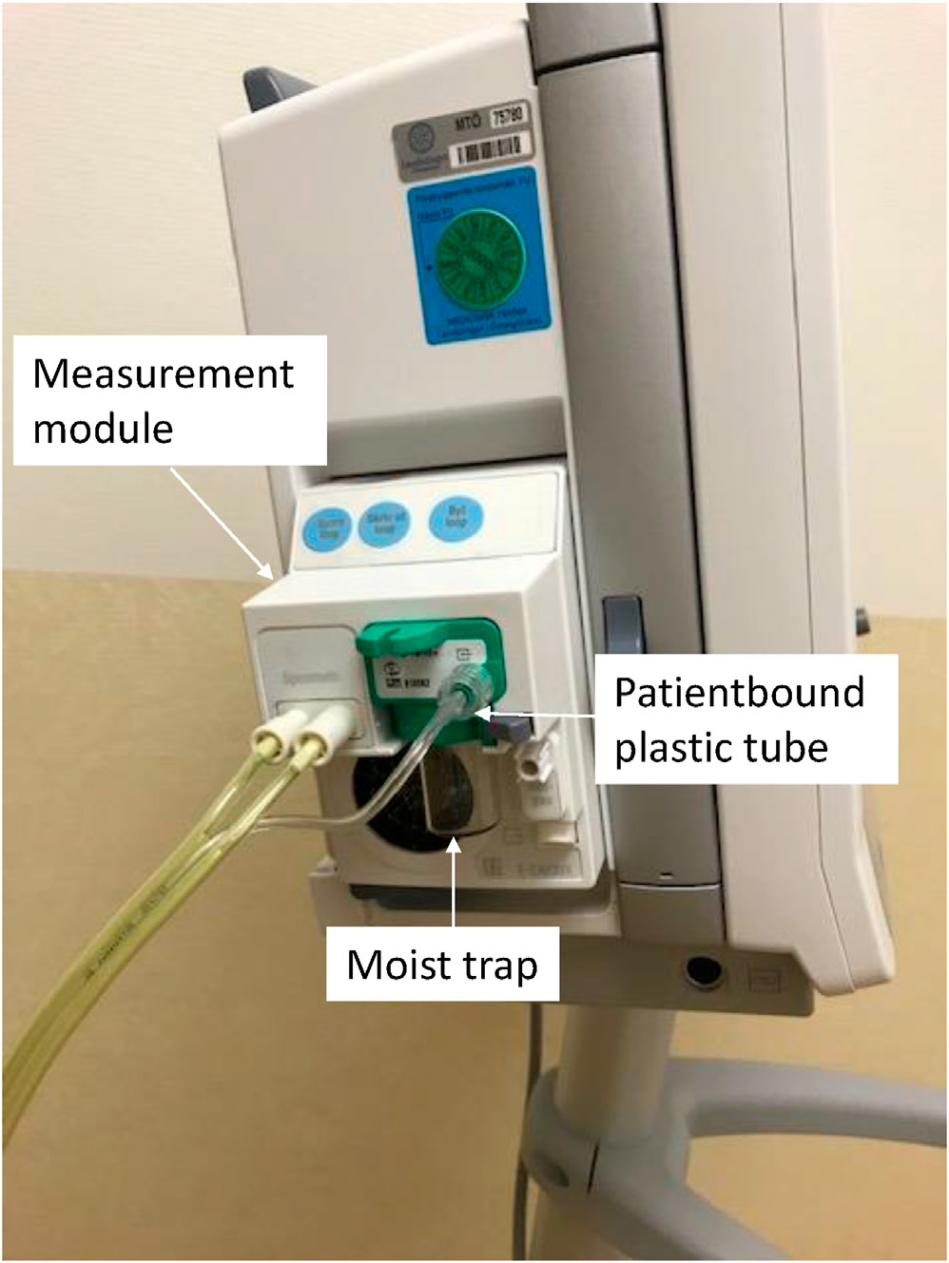
Photo of the involved calorimeter.

The three patients had two factors in common: a calorimeter and bronchoscopy. A calorimeter is a medical device that allows clinicians to accurately assess the energy expenditure in critically ill patients (Figure 2). Calorimetry was carried out every third day on the patients admitted to the ICU. According to routines, the moist trap (Figure 2) was changed once daily, whereas the plastic tube was patientbound (Figure 2) and stored in a plastic bag in the bedside table when not used. Patients 1 and 2 had used the calorimeter the very same day. In addition, bronchoscopy had been performed on all three patients. After each bronchoscopy, the bronchoscopes were cleaned, disinfected and hung on a stand in room temperature to dry.

Sampling was obtained from different sites of the calorimeter, vaporizers and plastic buckets used in respiratory therapy, the sink drain of all sinks located at the unit (in patient rooms, sluices, preparation room and wash rooms), and bronchoscopes. In addition, all patients admitted to the ICU were screened for *S. maltophilia* in samples from the lower respiratory tract. Cases were defined as patients with carriage of, or infection with, *S. maltophilia*. An additional inclusion criterion was that there had to be a connection in time and/or space with the index patient.

### Bacterial cultures

Environmental samples were collected with ESwabs (Copan Diagnostics Inc. Murrieta, CA, USA) and inoculated onto two different types of media for Gram-negative bacteria. The plates were incubated at 35 °C for approximately 48 h. Bacteria were identified to the species level with a MALDI Biotyper 3.0 (Bruker Corporation, Karlsruhe, Germany). The antibiotic susceptibility to trimethoprim-sulfamethoxazole was tested according to the recommendations of EUCAST (www.eucast.org).

### WGS

All *S. maltophilia* isolates from patients and the environment were subjected to WGS. DNA was prepared from a single colony of each isolate, using EZ1 DNA Tissue Kit (Qiagen, Germantown, MD, USA), with an included pre-heating step at 95 °C and a centrifugation step at 350 rpm for 15 min. Twenty ng of DNA was used for library preparation, using QIAseq FX DNA Library Kit (Qiagen, Germantown, MD, USA) with 8 min of fragmentation time. DNA libraries were sequenced on the MiSeq platform (Illumina, San Diego, CA, USA) with 2 x 300 bp paired-end reads, and the samples obtained an average sequencing depth of 82x.

Data analysis was performed in CLC Genomics Workbench v. 9.5.4 with the Microbial Genomics Module v. 1.6.2 (Qiagen, Germantown, MD, USA). Multilocus sequence typing (MLST) analysis was performed using the PubMLST (pubmlst.org) scheme for *S. maltophilia* [9,10]. Sequence data from previously unknown sequence types (STs) were submitted to pubMLST. Sequencing reads were mapped to the *S. maltophilia* NCBI reference genome NC_015947. Variants were called in relation to the reference genome with the following thresholds: frequency ≥ 90 %, sequencing depth ≥ 20x and quality (Phred) score ≥ 20 at the variant position and ≥ 15 in the ± 5 bp neighbourhood. Identified SNP positions were filtered based on a sequencing depth of ≥ 20x in all samples, a Z-value ≥ 1.96 and a pruning distance of 100 bp. The resulting 30 695 positions were then used to build a neighbour-joining phylogenetic tree based on the genetic distance between samples. Genomes were also assembled and searched for resistance genes using the ResFinder database [11], with thresholds of 98 % identity and 60 % length.

## Results

### Microbiological findings

Apart from the three patients infected with *S. maltophilia* in the lower respiratory tract, eight additional patients admitted to the ICU were screened for *S. maltophilia*. None of them were positive for *S. maltophilia*. However, one month later when the screening had been stopped, another patient (Patient 4) exhibited growth of *S. maltophilia* in a sample from the lower respiratory tract. This patient had been cared for in the same room as Patient 1 (Figure 1).

A total of 54 environmental samples were collected. Of these, 13 (24%) showed growth of *S. maltophilia*. To the culture-positive locations belonged two different sites of the calorimeter (the moist trap and the portal of the patientbound plastic tube, see Figure 2), the plastic buckets used during the respiratory therapy for Patients 1 and 3, sinks (*n* = 4) located in all four patient rooms, sinks in three of the sluices, the sink in the preparation room, and the sink in one of the washrooms (Figure 1). The bronchoscopes showed no growth of *S. maltophilia*.

All *S. maltophilia* isolates from the patients and the environment (*n* = 17) were susceptible to trimethoprim-sulfamethoxazole.

### WGS results

**Figure 3:**
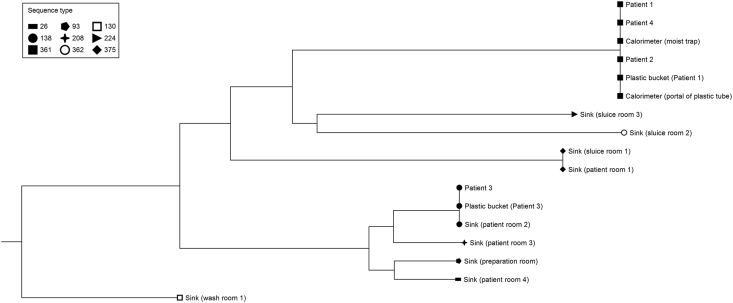
Phylogenetic tree based on single nucleotide polymorphism (SNP) analysis of whole genome sequencing (WGS) data from 17 *S. maltophilia* isolates. Node shapes represent sequence types (STs) based on multi-locus sequence typing (MLST). Two outbreak clones were identified: one belonging to ST361 (isolates differing by 0–2 SNPs) and one belonging to ST138 (isolates differing by 0–1 SNPs).

MLST and whole genome-wide phylogenetic analysis identified a high genetic diversity among the 17 *S. maltophilia* isolates collected from the ICU, and the distribution of the isolates was the same with the two methods (Figure 3).

MLST analysis identified nine different types among the 17 isolates, with a distribution that is in agreement with the SNP analysis results (Figure 3). Three of the STs were previously unknown. Two of these received novel MLST profile numbers (361 and 362) upon submission to the *S. maltophilia* pubMLST database [10]. The third type did not receive a profile due to the presence of two different nuoD alleles.

Two outbreak clusters were recognized (Figure 3). The larger one consisted of six isolates (35%) and belonged to the ST361 clone. Among these, the isolates from Patients 1 and Patient 4 were identical. They differed by one SNP to a group of three other identical isolates: those of a calorimeter site (portal of patientbound plastic tube), the plastic bucket located next to Patient 1 and the isolate from Patient 2. The isolates of Patients 1 and 4 also differed by a single but different SNP to the second isolate from the calorimeter (moist trap). In the smaller cluster, three isolates (18%) belonging to ST138 were included. Of these, the isolate from Patient 3 and from the plastic bucket located next to this patient, had identical SNPs, whereas the isolate from the sink located next to Patient 3 (in patient room 2), differed by one SNP. None of the isolates harboured a gene encoding resistance to trimethoprim-sulfamethoxazole.

### Control measures

Control measures were immediately taken, including screening of all admitted patients to the ICU and improved compliance to basic hygiene and cleaning routines. To limit bacterial growth in the water traps, every sink in the unit was rinsed once a week with boiling water. When rinsing inner cannula, a metal bowl was placed inside the original sink, and all contaminated water or other fluids were discharged in the sink located in the washrooms instead of in the patient rooms. In addition, no medical devices or items such as toothbrushes were allowed on the sinks or in their close vicinity to avoid contamination from aerosols. The plastic buckets used during respiratory therapy were replaced with single-use buckets, which were changed after each work shift. The moist trap and plastic tubes of the calorimeter were changed after each performed measurement.

## Discussion

In the present study, a minor outbreak of *S. maltophilia* at an ICU at Linköping University Hospital in Sweden was described. A total of four patients were involved, and two different clones of *S. maltophilia* were identified, ST361 and ST138. The ST361 clone caused the largest cluster and originated most likely from Patient 1, whose lower respiratory tract culture yielded growth of *S. maltophilia* prior to the admittance to the ICU. The clone was thereafter transmitted by a calorimeter, which had not been properly handled by the staff. According to the manufacturer's manual, the moist trap of the calorimeter should be changed after each measurement. Instead, this was carried out only once daily. Since the calorimeter was used every third day on the patients, several patients were at risk of being infected with *S. maltophilia*. The second cluster probably originated from one of the sinks in the unit.

Environmental cultures were collected from sink drains of every sink at the ICU and growth of different strains of *S. maltophilia* was identified in almost every room. The water traps of sinks constitute a wet and relatively protected environment, which favours the growth of bacteria and production of biofilms. The exposure to liquids and the waste discarded in the sinks may serve as a breeding ground for opportunistic and multiresistant bacteria that cannot easily be eradicated [12]. It has been described that sink drains in hospitals contain 10^6^–10^10^ colony forming units (cfu)/ml of bacteria of which approximately 10^3^-10^5^ cfu/ml are Gram-negative rods, especially waterborne bacteria [5]. These bacteria can infect patients via different transmission routes. There has been a clear increase in documented sink-associated outbreaks worldwide in recent years. However, few studies deal with the exact mechanism of transmission, i.e. from the sink to the hospitalized patients. In a study from 2017, mobilization of bacteria from biofilms in water traps of sinks to the surrounding environment was demonstrated by using green fluorescent-expressing *Escherichia coli* [12]. This was most likely the transmission route for the two last patients.

Replacement of contaminated sinks has been shown to reduce the infection rate in ICUs [13,14], but re-occurrence of growth have been described [15]. A more long-term solution would be to use sinks with a self-disinfecting function. There is already a product on the market that can disinfect the water trap [16], and similar products are needed to keep away an important source of not only carbapenemase-producing *Enterobacterales* and *Pseudomonas aeruginosa*.

Nosocomial outbreaks caused by *S. maltophilia* seem to be quite rare considering the low number of published articles. Several of them deal with airways and water in some form. In a study from 2013 [17], an outbreak of *S. maltophilia* at an ICU located in the United Kingdom was described and involved 23 patients (majority of the isolates from the respiratory tract), which were shown to belong to only two genotypes. Environmental sampling found the two outbreak strains in two sinks and in the drinking water of the cooling unit used for providing drinking water and mouth care to ICU patients. Likewise, a Spanish study reported a bronchoscope-associated pseudo-outbreak with 39 patients, highlighting the risks with contaminated medical devices [18].

Although outbreaks are relatively rare, the genetic relatedness of isolates in suspected outbreak situations needs always to be explored. A wide range of methods have been used through the years [19]. In this study, WGS was applied. It clearly showed the high genetic diversity among *S. maltophilia* in a single unit, which is in accordance with other studies [20,21]. Furthermore, it showed a dissemination of ST361. If this clone has features that render it more epidemic than others is not yet known, but with more use of WGS, certain clones may show themselves to be more prone to dispersal than others.

## Conclusions

An outbreak of *S. maltophilia* caused by two different clones and involving four patients in an ICU was confirmed by WGS. To our knowledge, this is the first study that demonstrates the involvement of a calorimeter in the transmission. The intervention was successful and no more patients were infected. To avoid transmission of *S. maltophilia*, which may cause serious infections in vulnerable patients, better attention needs to be paid to water sources and sinks located in hospital environments.

## Author contributions

We have contributed to the manuscript as follows:

MG: Design, acquisition and analysis of data, and drafting and revising the manuscript.

JW: Acquisition and analysis of data, and drafting and revising the manuscript.

AM: Design, analysis of data, and drafting and revising the manuscript.

We all approve of the submitted version, and there are no conflicts of interest.

## Conflict of interest statement

None.

## Funding source

This study was financially supported by 10.13039/100001424ALF from the Uppsala Region, Sweden. The sponsor had no involvement in the study.
